# Robot Assistance in Dynamic Smart Environments—A Hierarchical Continual Planning in the Now Framework

**DOI:** 10.3390/s19224856

**Published:** 2019-11-07

**Authors:** Helen Harman, Keshav Chintamani, Pieter Simoens

**Affiliations:** IDLab, Department of Information Technology, Ghent University—imec, Technologiepark 126, B-9052 Ghent, Belgium; keshav.chintamani@ugent.be (K.C.); pieter.simoens@ugent.be (P.S.)

**Keywords:** smart environment, Internet-of-Things, Internet-of-Robotic-Things, cloud robotics, IoRT architecture, ambient intelligence, continual planning framework, dynamic environment

## Abstract

By coupling a robot to a smart environment, the robot can sense state beyond the perception range of its onboard sensors and gain greater actuation capabilities. Nevertheless, incorporating the states and actions of Internet of Things (IoT) devices into the robot’s onboard planner increases the computational load, and thus can delay the execution of a task. Moreover, tasks may be frequently replanned due to the unanticipated actions of humans. Our framework aims to mitigate these inadequacies. In this paper, we propose a continual planning framework, which incorporates the sensing and actuation capabilities of IoT devices into a robot’s state estimation, task planing and task execution. The robot’s onboard task planner queries a cloud-based framework for actuators, capable of the actions the robot cannot execute. Once generated, the plan is sent to the cloud back-end, which will inform the robot if any IoT device reports a state change affecting its plan. Moreover, a Hierarchical Continual Planning in the Now approach was developed in which tasks are split-up into subtasks. To delay the planning of actions that will not be promptly executed, and thus to reduce the frequency of replanning, the first subtask is planned and executed before the subsequent subtask is. Only information relevant to the current (sub)task is provided to the task planner. We apply our framework to a smart home and office scenario in which the robot is tasked with carrying out a human’s requests. A prototype implementation in a smart home, and simulator-based evaluation results, are presented to demonstrate the effectiveness of our framework.

## 1. Introduction

The deployment of mobile and dexterous robots is being envisioned by the research community in an expanding range of environments. For instance, assistance and companion robots for elderly at home, and service robots in office buildings. These robots work alongside humans, and therefore must be able to adapt their task execution to unexpected changes in the environment’s state.

Instead of hard coding sequences of actions to accomplish a given task, a more generic approach is symbolic task planning, an artificial intelligence technique that can be applied to real-world robotics [1]. A task is formulated as a desired goal state, and planners autonomously find the appropriate set of actions (i.e., a task plan) to move from the current state to the desired goal state. Symbolic task planners are ran by continual planners, for example, References [2,3,4,5], which interleave sensing, planning and acting. The (re)planning phase is only performed when a state change that affects the plan is detected.

An increasing number of Internet-of-Things (IoT) devices with sensing and actuation capabilities are being installed, resulting in smart environments [6]. Embedding robots in smart environments, a concept referred to as the Internet-of-Robotic-Things [7], can extend the scope of tasks a robot’s continual planner can handle in two ways. First, the pervasive sensors provide valuable information, which can be incorporated in the planner’s estimate of the current world state. Second, actuation devices expand the set of available actions. For instance, imagine a robot that is able to drive around a house but cannot open doors, for example, because it lacks arms or is carrying an object. If a door on its planned path is closed, a robot that is not coupled to the smart environment will only discover the infeasibility of its current plan when the door is within the perception range of its own sensors. Whereas, an IoT door sensor could immediately notify the robot of the door being closed. If the door is also equipped with an remotely controllable door pump, the robot can request a smart home system to open the door. Otherwise, if there are no alternative routes available, a closed door would simply result in the robot failing to accomplish its task.

In this paper, we introduce our Hierarchical Continual Planning in the Now framework, which aims to (i) enable a robot’s planner to incorporate the state information and exogenous (off-board) actuation capabilities provided by IoT sensors and actuators; and (ii) reduce the frequency of and time spent (re)planning tasks in dynamically changing environments. Representing all devices directly within a symbolic task planner’s state, together with the sensor information and the actuation capabilities they provide, would cause unreasonable planning times. Therefore, in our framework, cloud-based state monitors inform the robot of IoT-sensed state changes that are relevant to its current task plan. Moreover, during the planning phase, remote devices are abstractly represented and calls to an external (ontology-based) module check if one or more devices are capable of executing an action.

Actions further along in the planning horizon are more likely to become unexecutable or unnecessary due to a unexpected change in the world’s state. State knowledge and actions are therefore split-up into a hierarchical structure. A single branch is planned and executed before the subsequent branch. This reduces how far in advance a detailed plan is generated and enables a robot to replan subsections of its task plan.

In Reference [8], we presented an early version of our planner that recreated the entire plan when an IoT or onboard sensor observations deemed the current task plan to be infeasible. In the current paper, we introduce a hierarchical approach, avoiding an entire replanning if an observed state change only impacts actions much later in the planning horizon. We have developed a much more generic mechanism that allows for ontology-based reasoning on IoT actuator capabilities. Moreover, results of deploying our framework in a smart home are presented. Our scenarios and experiments focus on robots providing assistance at home and in smart offices; however, our planning framework is applicable to any smart environment. Our framework does not address the challenges relating to multi-robot planning, for example, conflict resolutions and resource negotiations.

The remainder of this paper is structured as follows. A summary of related work is provided in Section 2. Background information on the concept of hierarchical planning in the now is provided in Section 3. Section 4 provides an overview of the framework architecture. Details on the different components are described in Section 5. Section 6 introduces how a engineer can design the hierarchy of state knowledge and actions. Finally, in Section 7 and Section 8 we evaluate our work through real world tests and simulated experiments.

## 2. Related Work

This section discusses several strands of related work. First, approaches that perform planning within a smart environment are introduced. This is followed by a discussion on how robots can reason on the capabilities of themselves and IoT sensors and actuators. The third subsection outlines how domain specific reasoning has been integrated into symbolic task planners. Finally, methods that reduce the amount of planning performed upfront are described.

### 2.1. Planning in Smart Environments

As explained in the introduction, the sensing and actuation capabilities provided by a smart environment can extend both the robot’s perception range and ability to manipulate the environment. Below, only works that incorporate IoT sensor and/or actuator devices into planners are discussed. For a broader survey on the integration of IoT and robotics, we refer to Reference [9].

In Reference [10], data from off-board RGB-D cameras allows a robot to create a plan that avoids congested areas. When a sensor nearby the robot detects congestion, the robots within the local area attempt to adapt their plan. If unsuccessful, a central scheduler recalculates the robots’ plans by reasoning on an ontology containing all robots, along with the tasks they can fulfil.

When using symbolic task planning in multi-agent robotics, which device (or robot) will execute an action tends to be explicitly stated within the plan, for example, MA-PDDL [11] and MAPL [12]. However, the number of IoT devices installed in smart environments is rising, and adding all these devices explicitly to the planning problem increases the planning time. This causes unreasonable delays before task execution can begin. Further to this, devices may become unavailable, causing the plan to fail; and new devices may be discovered, which could allow a less costly plan to be produced.

In Reference [13] plans containing abstract services are generated. These, abstract services, are mapped to actual devices during run-time. As the plan contains abstract services, replanning is not required when services appear and disappear. We aim to incorporate this concept into robot task planning by adding an abstract remote device to the planning problem. As we use a mobile robot, some knowledge about which device the robot should interact with is still required during part of the planning process. For example, if the robot requires an IoT coffee machine, it needs to know the location of the coffee machine.

The PEIS Ecology middleware [14,15,16] provides robots with the ability to discover what heterogeneous smart devices are available and subscribe to their capabilities for assistance with executing a task. When a device becomes unavailable, the ecology is automatically reconfigured to use an alternative device. Like PEIS, in our system a robot is responsible for planning its own task; however, rather than a robot directly communicating to devices, they communicate via a central server, which reasons about devices and their capabilities.

### 2.2. Capability Reasoning

Robots come with many different capabilities, for example, some may have the dexterity to open doors while others have no effectors for object manipulation [17]. A similar diversity in sensing and actuation capabilities is observed across smart environments. Some capabilities may be provided by more than one device. For instance, a door can be opened by another robot or by a nearby human. Moreover, the available functions of a device may evolve over time and, for example, become temporarily unavailable due to a malfunction. Therefore, during the planning process, the robot needs to know that there is at least one device (possibly itself) capable of performing an action for a given set of parameters (e.g., an open door action can be performed on door 1 but not on door 2); otherwise, the action should not be planned. We propose calling an external module, which queries the cloud for capable devices. The cloud reasons on an ontology about the capabilities of devices, and monitors their status.

Numerous robotic and IoT ontologies exist for reasoning about devices and there capabilities, such as IoT-O [18,19] and KnowRob’s SRDL [20]. These describe “things” and their relationships in the Web Ontology Language (OWL). Rather than coming up with another new ontology, our framework makes use of the KnowRob ontologies [21], which are part of the RoboEarth project. KnowRob enables robots to be described in detail (e.g., their software and hardware components) and each robot can be linked to one or more capabilities. As these capabilities do not include any parameters, we expanded this ontology (see Section 5.2.2).

In the RoboEarth project, if a robot has the required capabilities, an action recipe is downloaded onto the robot and tailored to its specific hardware to create an executable plan. Furthermore, a task plan is produced based on the most likely locations of objects. If the most likely location is incorrect, the next most probable location is used. When the object is detected, information is sent back to the cloud to update the probabilities. We aim to allow robots to execute tasks even if network connectivity is lost; therefore, in our system a robot is responsible for performing its own planning. Although a robot will be limited to its onboard capabilities, some tasks can still be accomplished.

In Reference [22], semantic maps are queried to enrich the planner’s initial state with additional knowledge about the environment. Providing the planner with all knowledge derived from the semantic map would be computationally expensive. Therefore, they only include knowledge relevant to the current context, that is, concepts stated within the goal. Moreover, “semantic-level planning” is performed to prune the initial state of unnecessary information. Similar to this approach, our framework only provides relevant information to the planner; however, our approach focuses on the splitting-up of actions and state into subtasks to reduce the amount to knowledge required by a task planner. Our approach also performs planning in the now, thus reducing the planning time required before the robot starts executing its task.

Köckemann et al. [23] integrated a constraint-based planner with an ontology to avail the capabilities of IoT devices. To accomplish this, they enabled ontology queries to be written within the planning language. Further, they also developed a method to generate the queries automatically from statements within the domain definition. Their examples and experiment focus on querying sensors. Similarly, we integrate a symbolic planner with an ontology but we focus on reasoning on actuation capabilities. We also develop a continual planning framework that enables the planner to be run onboard a robot, and the cloud to monitor IoT devices and reason on their capabilities.

### 2.3. Planners with External Reasoners

Often the (re)planning phase of a continual planner is performed by a classical symbolic (task) planner, for example, Fast Downward (FD) [24]. These planners take a symbolic representation of an agent’s behaviour and the world state as input, and search for a set of actions that will transform the initial state into the goal state. A popular symbolic language is Planning Domain Definition Language (PDDL). A PDDL problem definition usually consists of a problem file and a domain file. The problem file contains an initial (current) state and a goal state, both expressed using predicates, fluents and objects. Actions, along with their conditions and effects, are defined in a domain file.

As PDDL is restrictive in what can be represented, several works, such as TFD/M [17,25], enable external modules to be called during planning. These modules incorporate low-level domain specific reasoning into high-level symbolic planning to allow for better (i.e., more accurate and detailed) state estimation. For example, an external module could be a motion planner to discover if it is possible for a robot to reach a location [3] or a reasoner which determines if an object will fit into a container based on its shape and size [25]. Unfortunately, as external module calls increase the planning time, it can be difficult to balance determining the correct state with keeping computational costs down [4]. In our framework, which expands on the work by Dornhege and Hertle [4] and Speck et al. [3], an external module is called to reason on the capabilities of IoT actuators.

Checking the feasibility of plans with additional domain-specific algorithms has also been previously investigated for Hierarchical Task Network (HTN) planners. In HTNs, a domain file contains a set of compound and primitive (executable) tasks along with methods, which describe how to decompose each compound task into subtasks. Subtasks can consist of both primitive tasks and compound tasks (which will be further decomposed). HTN planners have been combined with algorithms for geometric task planning [26] and for resource scheduling [27]. When the lowest level of the HTN is reached, the respective algorithm is invoked and, if unsuccessful in finding a solution, backtracking is performed.

### 2.4. Interleaving Planning and Execution

Planning methods usually generate the full plan upfront before starting execution. Unexpected changes in the state of the environment can cause the plan to become infeasible; therefore, the effort to generate the full plan is wasted. As well as causing a cost to the robot itself, replanning can be costly to the system as a whole due to resources having been assigned to the plan (e.g., the use of external devices). Furthermore, additional state may become apparent during the execution of the plan, which can enable a more cost efficient plan to be generated. In this case, replanning is required to take this additional state information into consideration. To resolve these issues, scholars [28,29,30,31,32] have suggested planning short term actions in more detail than those later in the planning horizon.

Sukkerd et al. [28] propose multi-resolution temporal planning using a Markov Decision Process (MDP). Actions up until a given time are planned in detail; and those later in the planning horizon are planned at a coarser level of detail. Replanning of the coarser sections is performed with up to date state observation to generate a detailed plan. This approach enables distant actions to be taken into consideration when generating the detailed, short-term plan.

In Belief-Desire-Intention (BDI) approaches [33,34,35], an agent selects its intentions (i.e., plan) from a set of desires based on its beliefs about the current world state. Traditionally, the desires are a predefined library of plans [36]; however, BDI has also been combined with planners. For instance, Mora et al. [34] first select an intention and then plan the necessary actions. Besides actions, the plan can also contain intentions, which require further refinement. An agent revises its beliefs and triggers replanning when necessary.

Instead of time determining which actions are planned in detail, one can use the structure of hierarchical plans. HTN’s task decomposition can determine which high-level actions to plan in detail. Hierarchical Planning in the Now (HPN) recursively plans and executes actions [29]. An abstract plan is formulated, and the first composite action is planned and executed before decomposing the subsequent composite action. The authors use a hierarchical planning language in which the actions must be labelled with the level of abstraction, and the complete world state is always reasoned on. This has been expanded with belief states, that is, Belief Hierarchical Planning in the Now (BHPN), and planning using continuous state information (e.g., position) [30].

As our system builds on many of the concepts from HTN planners, it has many of the same benefits and disadvantages. Although HTN planners do not always guarantee an optimal plan [37], the use of compound tasks enables HTN planners to be faster [26] and simpler [38] than classical symbolic planning. HTNs are also claimed to represent tasks in a way more closely related to how humans think about problems [26,39]. Unfortunately, there is no standard planning language for HTNs and recent works, for example, Reference [40], attempt to transform a HTN planning problem into a classical (PDDL) planning problem to apply more advanced heuristics. Therefore, in our work, PDDL is split-up into a hierarchy.

In order to maximise the developer’s flexibility and to ensure compatibility with various types of smart environment, our planning framework differs from HPN [29] and BDI [34] systems in a number of ways. First, our approach splits-up both the state knowledge and actions into different layers; therefore, only knowledge relevant to the current layer and context is reasoned on. Second, any planner, including those using a different representation of the world, can be used at each layer. Third, by planning with different domain files at each layer, domains can be re-used in different scenarios and this prevents a single extremely large domain file from being developed. Finally, we also focus on how the planner can use IoT devices found within a smart environment and replan when a state change that causes the robot’s plan to be invalidated is detected.

## 3. Background: Hierarchical Planning in the Now

Our framework enables hierarchical task plans to be monitored, so that replanning is triggered on a sub-branch of the plan when IoT sensors detect unanticipated state changes. This background section describes the general concept of Hierarchical Planning in the Now (HPN), as introduced by Kaelbling and Lozano-Pérez [29]. In HPN, the initial task plan, generated by the planner, contains a sequence of actions that can be primitive or composite. Each of these actions will be executed in turn. The execution of a composite action will generate a sub-plan, which again will be comprised of primitive and/or composite actions.

**Figure 1:**
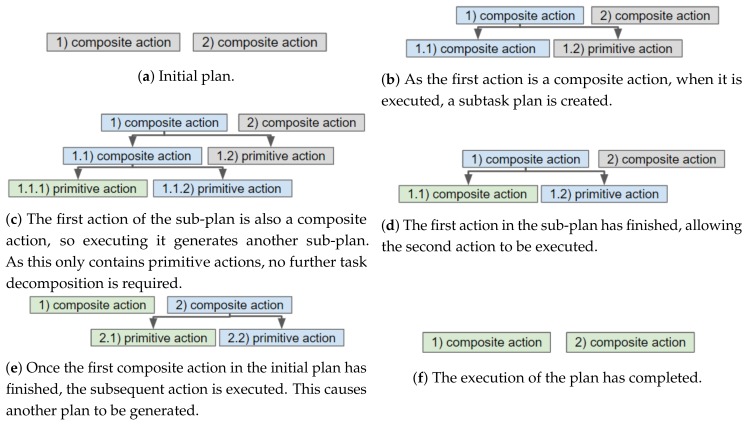
Grey boxes indicate actions which have not yet been executed; blue shows the actions currently being executed and green indicates executed actions. These figures describe the general concept of hierarchical planning in the now, introduced by Kaelbling and Lozano-Pérez [29].

This concept is depicted in Figure 1. In this figure, the initial plan consists of a sequence of two composite actions (Figure 1a). The first composite action produces a sub-plan composed of a composite and a primitive action (Figure 1b). As the sub-plan’s first action is also a composite action, it is decomposed and a plan containing only primitive actions is produced (i.e., Figure 1c). Once these primitive actions have been executed, the effects of composite action 1.1 are reached, enabling the next action within the second level to be executed (Figure 1d). This continues until all actions have been completed (Figure 1e–f).

## 4. Architecture Overview

**Figure 2:**
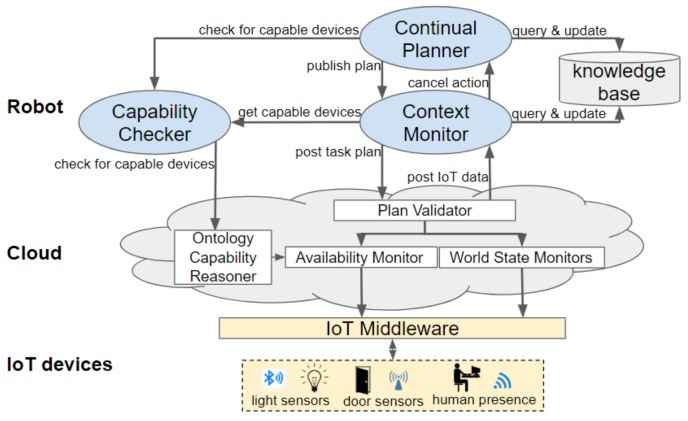
During planning, external module calls check if any device is capable of performing an action by calling a service running in the Capability Checker, which queries the cloud for capable devices and caches the results. The Context Monitor queries this when a plan has been generated, to know which device(s) should perform which action. The plan (containing the list of actual device) is sent to the cloud, which is monitoring the state of the environment and the devices. Any Internet of Things (IoT) data relevant to the robot’s plan is sent back to the robot and inserted into the robot’s knowledge base. If this information will cause the robot’s plan to fail, its current action is cancelled forcing the state to be (re-)estimated and replanning to be performed.

Our framework consists of components that are deployed onboard a robot and in the cloud. The architecture of the framework is visualised in Figure 2. This sections explains which components are deployed onboard the robot and which are run in the cloud. Further details, about these components, are provided in the subsequent section. Communication with the IoT devices of a smart environment is abstracted by an IoT middleware, namely, DYnamic, Adaptive MAnagement of Networks and Devices (DYAMAND) [41]. DYAMAND enables all heterogeneous IoT sensors to communicate using a standard format and IoT actuators can be commanded by sending JSON strings (via a central-server) over HTTP.

The main components onboard a robot are the Continual Planner and a knowledge base. The Continual Planner performs three processes: it composes the PDDL files based on the information stored in the knowledge base, it generates a task plan containing composite and primitive actions and it executes that plan. Running the planner on the robot allows it to continue operating when (wireless) network connectivity towards the cloud drops but requires limiting the amount of information transmitted from the smart environment to the robot. Sending the raw data streams of all IoT sensors in the environment would incur prohibitively large network bandwidth and associated battery drain. Moreover, the robot would lack the required computational resources to process raw data, especially if rich data from sensors such as cameras or microphones [42,43] is involved.

During the task planning phase, the Continual Planner will check via the Capability Checker if a remote (IoT) device is capable of performing an action. The Capability Checker queries the cloud-based Ontology Capability Reasoner for capable devices and caches the result. All reasoning over IoT actuators and their capabilities is thus delegated to the back-end components of the framework.

A robot, via its onboard Context Monitor, communicates its current hierarchical task plan to the cloud back-end. In the cloud, World State Monitors process the data produced by IoT sensors and the Availability Monitor tracks the availability of IoT actuators. The cloud informs the robot of unanticipated state changes affecting its plan and, subsequently, the robot decides if replanning is necessary. Rather than recreating the whole hierarchical task plan, replanning is triggered at the level containing the action(s) that will fail. As only a small subsection of the plan is replanned, the time spent planning is greatly reduced in comparison to planning everything upfront. If replanning fails, the composite action becomes invalid. This will trigger its parent’s (i.e., a higher-level’s) state estimation and replanning.

## 5. Incorporating IoT into Continual Planning

**Figure 3:**
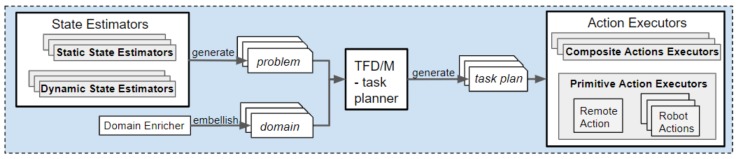
The continual planner calls the State Estimators (i.e., Static State Estimators and Dynamic State Estimators) and Domain Enricher to generate the PDDL problem and domain based on observations from the robot and smart environment. The TFD/M planner generates a task plan that contains primitive actions, to be executed by the robot and IoT actuators and composite actions. Composite action executors contain an instance of the the continual planner, which is configured with the relevant State Estimators, Action Executors and domain file.

Our continual planning component, shown in Figure 3, is an expanded version of the framework by Dornhege and Hertle [4]. The original framework loops through the phases of populating a PDDL problem by calling State Estimators, running the TFD/M task planner and then calling the appropriate Action Executor plug-ins to translate actions into executable robot instructions. We have expanded this framework to incorporate IoT sensed information, utilise the capabilities of IoT actuators, produce a hierarchical task plan and monitor that task plan. This section first discusses how the PDDL files are formulated. Second, the capability checking process, which is performed during the search for a plan, is described. Subsequently, the context monitoring and replanning processes are introduced. In the final subsection, the action execution is detailed.

### 5.1. Generating a Planning Problem

In the first phase a planning problem is generated by a set of State Estimators, which populate a PDDL problem file and a Domain Enricher, which populates a PDDL domain file. These files can then be provided to a task planner (in our implementation, this is TFD/M) to produce a task plan containing both primitive and composite actions. Each action will be executed by its corresponding Action Executor (see Section 5.4.2). Which State Estimators, Action Executors and domain file should be loaded, is stated within a configuration file. Each composite action, that is, branch of the hierarchy, can be configured with its own domain file and instances of State Estimators and Action Executors. State Estimators and the Domain Enricher are detailed in this section.

#### 5.1.1. State Estimation

The PDDL problem file contains a goal state and the current state. The problem file is populated by calling a set of State Estimator plug-ins, each of which implements a fillState method. State Estimators query the most recent state either directly from the robotic middleware (e.g., to gain the robot’s position) or from the knowledge database and transform this information into PDDL statements. The knowledge base, implemented in MongoDB, stores information on the current state and the goals. Updates to the knowledge database come from external IoT devices via the Context Monitor and from the result returned by Action Executors.

We categorise State Estimators as either static or dynamic. Static State Estimators are called once, and insert immutable state information such as waypoints and immovable objects. Dynamic State Estimators are called at the start of each iteration to update the problem file; for instance, to reflect a change in the current state or to add a new goal. Table 1 presents an overview of the different State Estimators in our system; further State Estimators can easily be added.

#### 5.1.2. Defining Actions and Devices

The PDDL domain file contains the set of action definitions from which the TFD/M planner generates a task plan. In traditional approaches, this list is predefined with a fixed set of actions and the same domain file is reasoned on in every iteration. In realistic environments, this list can quickly grow in size, for example, for each type of object a robot may encounter different manipulation actions could be defined.

Instead of always providing an exhaustive and predefined list of actions to the TFD/M planner, a Domain Enricher is called. The Domain Enricher analyses the PDDL problem, obtained after calling the State Estimators and expands it with actions that are relevant to the defined entities/objects. Starting from a minimal domain file, only containing the most elementary actions a mobile robot can execute (e.g., for a navigation layer this includes actions to drive around the environment and inspecting objects), further actions are inserted in subsequent planning iterations. For instance, if a closed door is detected on the robot’s path, the sensed_obstacles State Estimators will add a door to the problem file and the Domain Enricher will insert the open door action definition, which is shown in Listing 1. Similarly, if the robot discovers a box, a push_box action will be inserted into the domain file. These actions are obtained by requesting all actions that can be performed on the object type, for example, objects of type door, from the cloud (which, for each object type, has a file containing the set of action definitions).


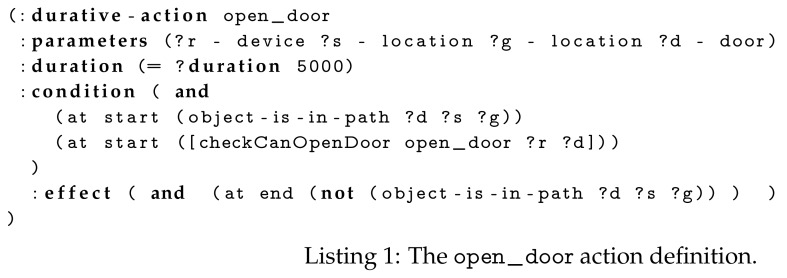


The actions provided by IoT devices are also defined in the domain file. Since the continual planner must be able to request a device to execute an action (e.g., the robot itself, a door pump, an IoT light switch or another robot), one solution would be to declare all individual devices as objects in the PDDL problem file. Due to the resulting expansion of the problem file, this solution would lead to long planning times. We recall the reader that frequent replanning might be required in dynamic smart environments. Rather than defining all IoT devices in the PDDL problem file, we introduce a single “remote” object that represents all remote devices.

### 5.2. Capability Checking

The availability of remote devices and thus the actions they can perform, may change over time. An IoT device may become unavailable for numerous reasons, for example, because it is being used by another service (or human), is under maintenance, is low on battery or has a degraded network connection. As some actions can be performed by more than one device, this does not necessarily mean the action cannot be executed. For instance, a door can be opened by an electric pump, another robot or a nearby human; and a cup of coffee can be fetched from another location if the nearest machine has run out of coffee beans.

During the search for a plan, the TFD/M planner will evaluate the conditions of an action many times with different argument combinations. It is difficult to formally represent the knowledge that is needed to reason on the availability of an action in PDDL. Therefore, our framework provides an external module, which in turn leverages an ontology-based reasoner, deployed in a cloud back-end. In this section, the robot’s capability checking module and Capability Checker are described, followed by the cloud’s Ontology Capability Reasoner.

#### 5.2.1. Capability Checking Module

The TFD/M task planner of Reference [25] enables external modules to be called during the planning phase. These enable domain-specific code to be executed. Inside the PDDL domain file we define a list of external module definitions. Like predicates, these are stated within the conditions of actions and are checked during the search for a plan, but rather than being compared to the world model, external code is called. In our case, this is a single capability checking module.

The first two arguments in the capability checking condition are always the name of the action and a device, that is, the name of the local robot or “remote”. The name of the action is given so that, after a plan has been found, a planned remote action can be associated with the discovered (capable) remote device(s). The number and type of the remaining arguments are specific to the action. For instance, in the example of Listing 1, checkCanOpenDoor has a parameter of type door; whereas checkCanFillObject would have two parameters, for example, a cup and coffee. If the name of a robot is passed (as the second argument), the capability checking module will evaluate based on the robot’s current capabilities. If “remote” is passed, the module will communicate with the Capability Checker, which queries the cloud for a list of capable devices and caches that list. The cache is queried for subsequent calls to speed up the process. Once a branch of the plan has been executed, the cache is cleared for actions within that branch (layer). This allows any newly available devices to be reasoned on in subsequent branching. In the cloud back-end, ontology reasoning determines the list of remote devices that can execute an action.

#### 5.2.2. Ontology-Based Reasoning

**Figure 4:**
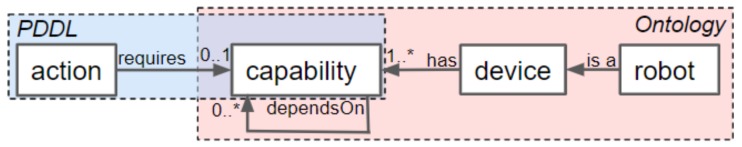
Outline of what we define action, capability, device and robot as. PDDL actions require a capability and devices along with what they are capable of are defined in an ontology.

To decouple the availability of an action from the availability of a specific device, the requirements of each action are modelled as a set of capabilities and each device has a number of capabilities. These relationships, depicted in Figure 4, are inspired by the SRDL ontology [20]. We have expanded the ontology by allowing capabilities to have requirements (e.g., the OpenDoorCapability can require a door to be specified) and an associated cost. The cost allows the planner to select the least costly device to perform an action. Currently this is a static (manually defined) value; in the future we envision more intelligent reasoning and learning being utilised. Moreover, we created a device class which represents simple devices, such as electronic doors, IoT lifts and smart coffee machines, as well as (mobile) robots defined in SRDL [20].

Upon receiving the request for capable devices, the ontology is queried for devices with a capability of the required type, for example, for all devices that have a capability of type OpenDoorCapability. The OpenDoorCapability has a “canOpenDoor” property matching a specific door; if this property is missing the device (or human) is assumed to be able to open all doors. The Ontology Capability Reasoner also queries the IoT Context Monitor to check the device is available (e.g., is not down for maintenance). All capable devices, along with the costs associated with the capability, are returned to the robot.

### 5.3. Context Monitoring

After a (hierarchical) task plan has been formulated, context monitoring and plan execution (see Section 5.4) occur in parallel. Context monitoring is the process of continuously evaluating the sensor data received from IoT devices, as well as the status of the devices and identifying any possible state changes that affect the current plan. If replanning is necessary, this is performed at the layer of the hierarchy affected by the state change. The robot’s onboard Context Monitor is discussed, followed by the cloud-based Plan Validator and IoT context monitors (i.e., Availability Monitor and World State Monitors).

#### 5.3.1. Robot’s Onboard Context Monitor

The Context Monitor, running onboard the robot, is the component responsible for keeping track of the hierarchical task plan and triggering the replanning of a branch of the plan. When a plan has been generated, it replaces all references to the generic “remote” object with a list of actual devices, which it obtains from the Capability Checker. The full hierarchical task plan, containing the device list(s), is then announced to the cloud-based Plan Validator. The Context Monitor will receive relevant IoT state updates from the cloud.

Although performing planning in the now restricts how far in the future a detailed plan is produced, state changes can still cause parts of the plan to become infeasible. The robot itself may detect that its current action will/has failed, for example, a box is blocking its path when executing a drive_base action; or an IoT device may detect state that will cause one or more of the robot’s planned actions to fail, for example, a door closes. In both these cases the robot needs to replan, to either bypass the issue (e.g., find a different route) or solve it (e.g., open the door). When a state change that violates one or more preconditions of a planned action occurs, the state change is inserted into the robot’s knowledge base and the executing action from the affected layer is stopped, along with its subtasks. For instance, in Figure 1c, if primitive action 1.2 will fail, composite action 1.1 (as it is currently being executed) is stopped. Thus, primitive action 1.1.2 is also stopped. This will trigger composite action 1 to replan its subtask.

In other situations, a remote device may become unavailable. If there is an alternative device available and all necessary dependencies are still met, no replanning is required. This is because the actual device that will execute a remote action is only resolved by the continual planner when the execution of a remote action is started.

Even when an alternative device is present, it might still be necessary to trigger a replanning. This is the case if another action depends on the choice of the remote device. For instance, in the plan: (1) move_to_object(rob1 remote), (2) hand_over_object(rob1 cup remote), (3) fill_object(remote cup coffee), where “remote” will be replaced by [coffee_machine1, coffee_machine2], the actions prior to the remote action (i.e., fill_object) mention the remote device. Therefore, if the execution of one of these actions has started and the initially selected remote device (i.e., first device in the list) malfunctions, replanning is required to enable the robot to move to the alternative coffee machine. Note: if remote actions have dependencies, remote actions that are executed by different devices should be planned within different branches of the hierarchy (e.g., the robot should move to and use one device before planning its navigation to and use of, a second device).

#### 5.3.2. Plan Validator and IoT Context Monitor

The Plan Validator communicates with robots and keeps a list of each robot’s plan and the objects/devices whose state could affect that plan. The IoT context monitors, that is, an Availability Monitor and set of World State Monitors, receive messages from the IoT middleware and provide the object specific information the robot requires when its plan is affected by a state change. This section describes the Plan Validator, followed by the Availability Monitor and World State Monitors.

The Plan Validator receives the (JSON) messages sent by one or more robots’ Context Monitors. These contain the robot’s IP address and hierarchical task plan in which “remote” has been substitute with the list of capable devices. The Plan Validator parses the task plan. Then queries the IoT context monitors for the set of objects (e.g., doors, cups, boxes, etc.) and devices, whose state could affect the robot’s planned actions. If the current state of an object or device already affects the robot’s plan, the robot is instantly informed. To receive the state changes, the Plan Validator registers itself as an observer of the Availability Monitor and each of the World State Monitors (which are observables). When informed that an object/device has changed state, the Plan Validator notifies the appropriate robots. The notifications, sent to the robot(s), contain which action the state change affects and the information provided by the monitors. Although the Plan Validator handles multiple robots’ plans, resource negotiations are beyond the scope of this paper.

When initialised, the Availability Monitor and World State Monitors query the ontology for the list of objects they should monitor the state of, that is, the Availability Monitor gets all objects of type device and, for example, a Door World State Monitor gets all objects of type door. To determine which objects a robot should know the state of a default method is provided, which simple checks the monitor’s list of objects against the robot’s planned actions’ parameters and returns the matches. In some cases, some domain specific processing is required. For example, the Door World State Monitor checks the plan for drive_base actions and returns a list of doors that sit between two locations the robot plans to drive between (these locations are also stated in the ontology).

When the IoT middleware (i.e., DYAMAND), announces a state change (i.e., posts a JSON string, which contains the name of the object/device and its state) the relevant monitor consumes that message. The monitor then provides the Plan Validator with the information that should be sent to the robots, whose plans could be affected by the change. In the case of the Availability Monitor, this information just contains the name of the device and its availability (i.e., a boolean value). For the World State Monitors, this includes the objects and PDDL statements that should be inserted into the problem file. For instance, the Door World State Monitor provides a PDDL statement declaring which locations the door blocks the robot from driving between, for example, (object-is-in-path door1.1 doorway1.1_room1.1 doorway1.1_room1.2).

### 5.4. Plan Execution

**Figure 5:**
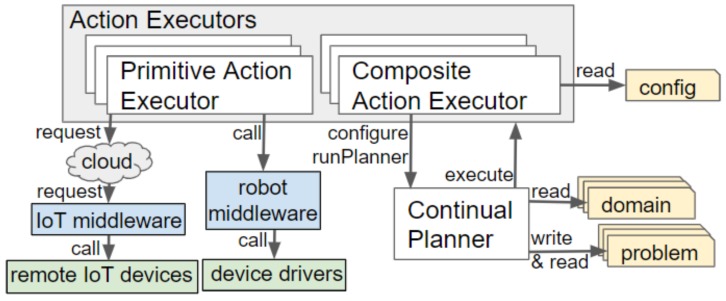
Functionality of the types of Action Executors. Grey boxes indicate types of plug-ins, white indicates classes/instances and yellow shows files.

Each action, defined in the PDDL domain hierarchy, is linked to an Action Executor, a plug-in containing the logic and low-level instructions to be executed. These plug-ins implement a execute() method, which is called by the continual planner. The workings of this method depend on whether the Action Executor is categorised as a Primitive Action Executor or a Composite Action Executor. Examples of both types are provided in Appendix A. Figure 5 illustrates how these interact with the other components of our framework.

#### 5.4.1. Primitive Action Executors

Primitive Action Executors perform the lowest-level actions, for example, open_door, drive_base, request_lift. These include Local Action Executors and a single Remote Action Executor. Local Action Executors interact with the robot’s actuators and sensors through a robot middleware, for example, Robotic Operating System (ROS) [44]. For example, the drive_base action will be executed by the DriveBaseActionExecutor, which, after querying the knowledge base for the geometric position of the symbolically represented location, interacts with the move_base ROS ActionLib to command the robot to drive to a specific position.

Any actions involving remote (off-board) devices are delegated to a single Action Executor that acts as a proxy for all IoT actuators. When remote action execution is required, the RemoteActionExecutor will retrieve the (least costly) device to execute the action from the Context Monitor. The action request is then sent to the cloud back-end (via a blocking HTTP post), where it is redirected to the appropriate actuator service via the IoT middleware.

#### 5.4.2. Composite Action Executor

Further planning is required for high-level (abstract) actions in order to create a plan containing only primitive actions. When invoked, a Composite Action Executor will start a new instance of the continual planner and configure it with the necessary domain file, State Estimators and Action Executors. If the resulting plan also contains composite actions, the procedure is repeated. Using this recursion, the execution of a composite action, ultimately, results in a set of calls to Primitive Action Executors. We provide a base CompositeActionExecutor class, which performs the action execution and includes 4 overridable methods: (i) to load the correct plug-ins and domain file (which, by default, are read from the configuration file), (ii) set the goal state, (iii) handle successful completion and (iv) handle failure.

A threshold on the number of sense-plan-act iterations a composite action will perform can be set to prevent endless attempts. Performing multiple iterations enables any additional (evolving) state to be taken into consideration. For instance, if a human crosses the path of the robot, the robot’s drive action might temporarily fail. By preforming a sense-plan-act iteration, the robot can continue driving after the human has moved. When the threshold is reached, the cloud back-end is notified. This enables the task to be assign to an alternative robot. In the future, this can enable the system to learn about how successful composite actions are likely to be given the current context.

## 6. Designing the Hierarchy for Smart Environments

An important aim of our framework is to reduce the frequency of replanning and, when replanning is required, reduce the time spent replanning. Applying principles of hierarchical planning, the domain knowledge is split-up across multiple re-usable domain files, each containing action definitions written in PDDL. Moreover, only knowledge relevant to the current context is inserted into the PDDL problem file. Actions in higher levels of the hierarchy are more generic and abstract the details of the lower level actions. This section describes the design process performed to create the layers of the hierarchy for our experiments.

**Figure 6:**
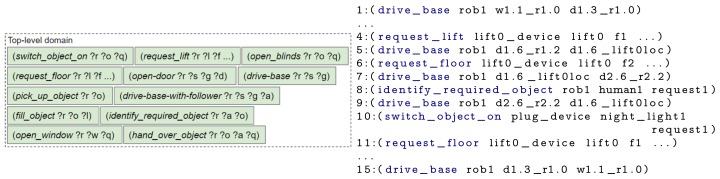
All actions definitions are contained within a single domain file. When planning everything upfront, the planner must make an assumption on the most likely request and include all actions needed to fulfil that request. In all figures the following words are abbreviated: doorway is shortened to d, waypoint to w, room to r and floor to f. In all proceeding figures blue boxes (with downwards arrows) indicate composite actions and green is used for primitive actions.

**Figure 7:**
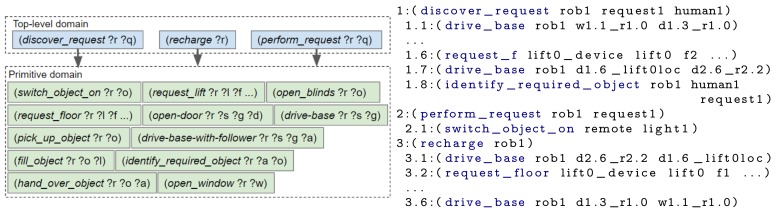
Action definitions split-up into two domain files; and executed actions when the state and actions are split up by key concepts.

**Figure 8:**
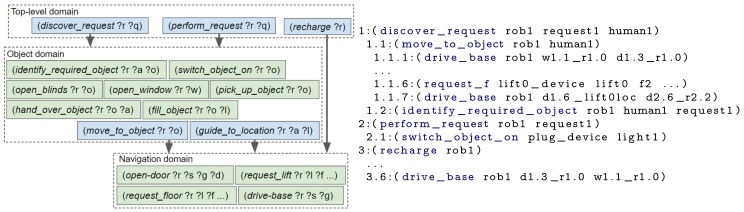
Action definitions split-up into three domain files. Executed actions when repeated groups of actions are separated.

**Figure 9:**
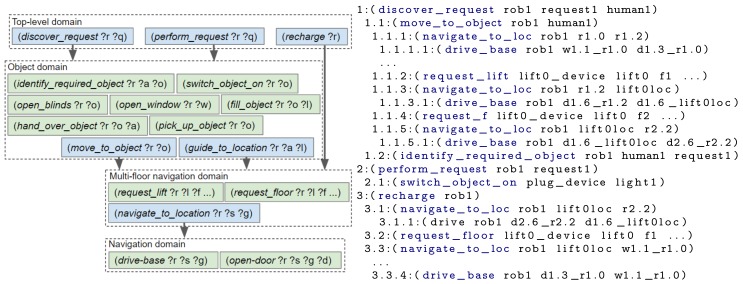
Domain files and executed actions for when we have split-up knowledge about what floor the robot is on. The fully hierarchical approach.

First, an example scenario set in a smart home is introduced, followed by the drawbacks of not using a hierarchy. The last three subsections describe the three ordered steps an engineer could take to design the hierarchy: (i) splitting up key concepts, (ii) separating repeated blocks and (iii) reducing unused state knowledge. The actions contained within each domain file, for each design step and a plan created using the domain file(s) are shown in Figure 6, Figure 7, Figure 8 and Figure 9. To improve legibility for the reader, figures show only the actions relevant for our discussion. The full plans are provided in Appendix B. As previously mentioned, a description of all composite and primitive actions in our final domain files is given in Appendix A.

### 6.1. Example Scenario

A smart house, with two floors and an IoT-controllable lift, is notified via a bed sensor that a child steps out of bed when they are supposed to be sleeping. The child may have woken up because, for example, it is too hot, they want the night light on or they are thirsty. Initially the robot is at its charging location and, when the child gets out of bed, the robot is instructed to complete the child’s request. The robot must drive to the bedroom, find out what the child wants, carry out their request, which is to switch on a night light and then return to the nearest charging station to await further instruction. Below, this scenario is worked out.

Moreover, to show the applicability of our system to different situations, our domain files also contain actions needed to guide a visitor and to fetch a cup of coffee. As well as being applicable to a smart home, these are applicable to a smart office building.

### 6.2. Baseline: All Actions and State within a Single Level

Without a hierarchy, a planner would need to plan in terms of very specific low-level actions, a few examples are shown in Figure 6. When the goal is provided to the planner, the entire plan is generated. Therefore, if a state change causes the plan to become invalid (e.g., a device becomes unavailable or a door in the robot’s path closes), the robot must replan the entirety of its plan. As the state may change again prior to the affected action being executed, the time spent (re)planning may be deemed unnecessary. For our example scenario, the Listing in Figure 6 shows the plan generated by the TFD/M planner. In the generated plan, after discovering the child’s request, the robot starts driving towards the location of its charging point (action 9) before performing the user’s request to switch on the night light (action 10). More natural behaviour would be to switch the night light on first. This could have been amended by creating a atom that prevents the robot from driving while fulfilling a request; however, we opted to not add this atom as to fulfil a request the robot might need to drive.

### 6.3. Splitting up Key Concepts

The main disadvantage of the above approach is the requirement to replan when a different request is made or the state of the environment changes. The chance of replanning can be reduced by adding a layer of abstraction onto the primitive actions. At a high-level the scenario has three main subtasks: (i) discovering what the request is, (ii) performing the request and (iii) returning to the charging point. Hence, a first step towards the design of our hierarchy is to create two levels, with the three generic actions in the top level and specific actions in a primitive domain. The resulting domain files are represented in Figure 7 alongside the actions that would be executed, that is, the composite and primitive actions that were planned by running our framework.

### 6.4. Separate Repeated Blocks

There are sets of (composite and/or primitive) actions which will be planned by many different composite actions, for example, navigation and object manipulation. We separate these actions into different domain files, to allow them to be used by multiple composite actions. In our example (see Figure 8), we created separate domain files for actions related to object interaction and actions that enable the environment to be navigated. By performing this separation, the range of tasks the robot can perform can be expanded without having to either repeatedly re-write the PDDL for navigation or continually add actions to a single increasingly complex domain file.

### 6.5. Reducing Unused Knowledge

When the number of objects within a problem file is increased, an exponential increase in the planning time is observed. This is due to the state explosion problem [45,46]. The fewer objects included within the PDDL problem, the fewer possible states and therefore state transitions, the planner has to search through. We aim to minimise the number of objects within the problem by only inserting those relevant to the robot’s current context or are likely to soon appear within the robot’s plan. This is achieved by splitting up more distant and loosely related objects. For our example, we split the navigation state knowledge up based on what floor of a building the robot is currently on. If the robot is on floor 1, it does not need to know the details (e.g., rooms and waypoints) of floor 2 until it reaches that floor. The domain and resulting plan are shown in Figure 9.

To provide an alternative example, which uses the types of objects rather than their location, a factory domain is described. In a smart furniture factory, a robot could be requested to construct a table and a chair. When making a table the robot does not need to know the state of the parts and tools required to make the chair. Thus, rather that reasoning over all objects and actions, the construction of the chair and table can be split into two separate branches.

Further splitting up of our hierarchy could be performed (e.g., navigation separated by room), however, as each level needs to be planned adding more levels causes unnecessary additional computational costs. If there is no clear separation and two actions/sub-goals are greatly dependent on the state of an object, these actions should not be split-up. Initially, designing the hierarchy may take more time than writing a single PDDL domain file but these layers are reusable.

## 7. Proof of Concept in a Smart Home

We developed a proof of concept in our HomeLab (https://www.imec-int.com/en/homelab) (smart house) with a Pepper (humanoid) robot. The HomeLab is a two-story house (600 m^2^) equipped with sensors and a home automation system, which communicates via DYAMAND [41]. The components onboard the robot were developed as nodes for the Robot Operating System (ROS) [44]. The robot used in our proof of concept has very limited dexterity, that is, is unable to carry or manipulate objects. For robots with greater dexterity, rather than just navigating the environment and commanding IoT devices, further tasks could be included, for example, the option of making and delivering a drink (see Section 8.2 for a simulated coffee fetching scenario).

**Figure 10:**
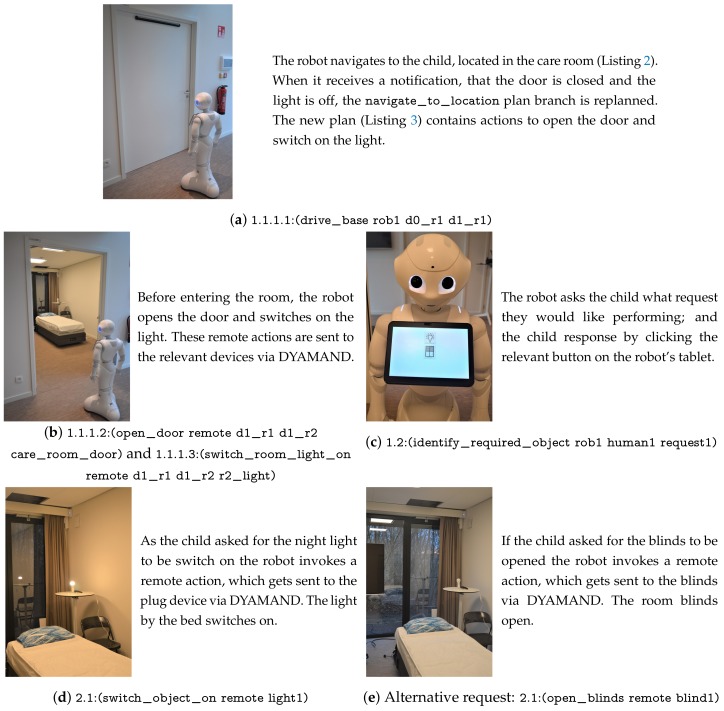
Real world tests. The full list of PDDL executed actions are given in Listings 3 and 4. Doorway has been shortened to d and room to r.

A similar scenario to that described in Section 6.1 was setup. In this experiment the child is located in a room on the same floor as the robot and the robot requires the room door to be opened and the room light to be switched on before it can enter the room. This experiment shows how IoT devices enable a robot to complete a task it would otherwise be incapable of. Photographs alongside a description of the actions the robot is performing are shown in Figure 10 and videos have been provided as Supplementary Material. This section describes the steps taken by our framework, when the hierarchical structure of Figure 9 was provided as input and the robot’s goal was set to (completed request1).

Initially, the robot’s navigate_to_location planning branch only knew the static map (walls and waypoints) and thus generated a plan containing two drive_base actions, see Listing 2. The full (hierarchical) task plan was submitted by the robot’s Context Monitor to the Plan Validator in the cloud. The cloud (i.e., the Door State Monitor and Light State Monitor) reasoned on the path in the plan and realised the robot required the room door to be opened and the room light to be switched on. As this state invalidated the current plan, the Plan Validator sent this information to the robot’s Context Monitor. The Context Monitor preempted (i.e., stopped) the drive_base action although the robot by itself reported no failures (since it had not arrived at the closed door yet).


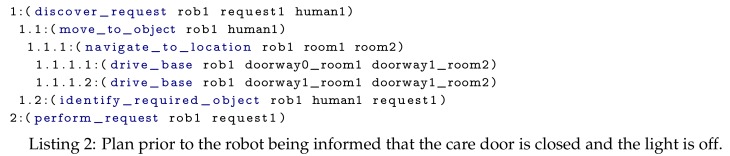


Only the navigate_to_location branch of the plan was modified by this cancellation. At the start of a new iteration of the continual planner, the sensed_obstacles State Estimator inserted the door and light objects into the PDDL problem. As replanning was required, the Domain Enricher immediately loaded the open_door and switch_room_light_on action definitions.

Using the Capability Checker, the robot’s planner determined that the robot itself did not have the capability to open doors or switch on lights. Therefore, without the assistance of IoT actuators the robot would be unable to complete its task. In our ontology there are two devices capable of opening the door: the IoT enabled door actuator and a human. Once a plan (i.e., Listing 3) had been generated, the Context Monitor retrieved the devices that can perform the remote actions from the Capability Checker. Subsequently, it replaced the “remote” objects in the plan with an ordered list of these devices, then sent the plan to the cloud. Capable devices are ordered by their static cost, which was manually defined in the ontology (i.e., the human’s capabilities were set as more costly that the IoT devices’). If a device within the plan becomes unavailable the cloud informs the robot, which would then use the alternative device. Since it is less costly to request the IoT enabled door actuator to perform the action, the RemoteActionExecutor asked this device to perform the open_door action. These steps are the same for the room light but the remote action request was sent to the IoT light switch.


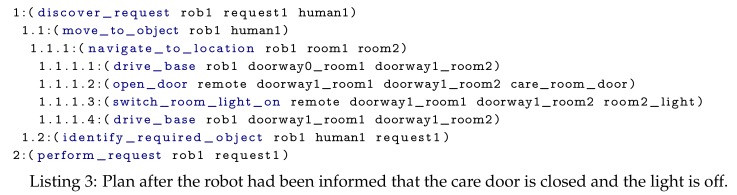


Once the robot entered the care room the child pressed a button on its tablet, which identified the request they wanted performing. The perform_request action was then executed. Depending on the child’s request this either generated a sub-plan to switch on the night light (see Listing 4) or to open the blinds. During the experiments, both these actions were performed by remote devices.


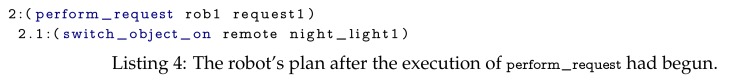


## 8. Simulated Experiments

**Figure 11:**
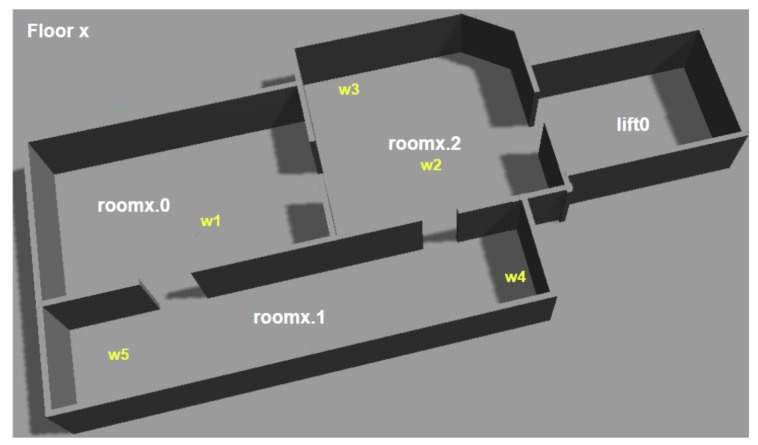
Gazebo simulated world used for the experiments. We use an extended version of the world originally created by Speck et al. [3]. Waypoints (e.g., w1) that are used during the different experiments have been indicated on the map. For all experiments there are also waypoints either side of doorways.

Through simulated experiments we aim to demonstrate (i) how much representing all devices as a single “remote” device has reduced the number of calls from the TFD/M planner to the Capability Checker; (ii) our framework’s ability to trigger replanning when a device becomes unavailable, (iii) the effect increasing the number of objects in the environment has on the planning time and (iv) the effect increasing the number of layers in the PDDL hierarchy has on the planning time. All these experiments use an environment simulated with Gazebo, this is shown in Figure 11. The State Estimators and Action Executors used are described in Table 1 and Appendix A, respectively; the goal the goal_creator sets is specific to each experiment. Each subsection describes the experiment set-up, followed by the results.

### 8.1. Exp. 1: Single “remote” Object to Represent all Devices

In this section, experimental results are presented to show how including all remote devices as separate objects in the PDDL problem compares to representing the remote devices as a single object. The number of calls from the TFD/M planner to the capability checking module, for a varying number of devices, are provided in Table 2. The total number of states generated by TFD/M, during its search for each (sub)plan, is also provided. Further details on TFD/M are provided in References [25,47].

#### 8.1.1. Set-Up

In this experiment the robot is tasked with simply navigating to another room on the same floor (i.e., from w1 in room1.0 to room1.2). Along its way, the robot must open a door and switch on a light. An initial plan without involving doors and lights is generated and sent to the Plan Validator, which informs the robot that the door is closed and the light is off. This causes the robot to replan and thus create a plan that includes opening the door and switching on the light.

The list of executed actions when there was a single “remote” object is shown in Listing 5. When all devices were stated within the problem, the same plan was produced but instead of “remote” the actual device name was stated in the plan. For this experiment, we simple increased the number of devices in the ontology and, when all devices were represented, these were also inserted into the PDDL problem file.


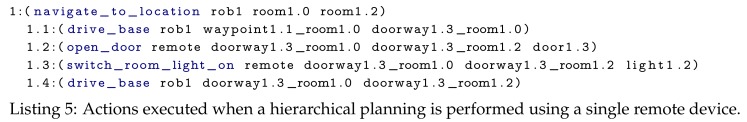


#### 8.1.2. Results and Discussion

The results of this experiment are presented in Table 2. When all devices were stated in the problem, as the number of devices is increased so did the number of calls to the capability checking module and the number of generated states. Whereas, when a single remote object was stated no increase was observed. For each of these experiments, the onboard Capability Checker only performed 2 HTTP requests to the cloud-based Capability Reasoner, that is, one for each of the remote actions (the open_door and the switch_room_light_on actions), as the Capability Checker caches the list of capable devices.

### 8.2. Exp. 2: IoT Device Failure and Plan Quality

Our second set of simulated experiments employed a scenario in which an IoT actuator, that a robot must navigate to before using, became unavailable. The quality of the executed plan (i.e., number of primitive actions) and the time spent planning are discussed. All experiments were ran on a virtual machine with 9GB of RAM and an Intel i7 2.9GHz CPU. The scenario also demonstrates the applicability of our designed hierarchy to a different situation, that is, a smart office environment. Further, these experiments demonstrate that our framework is able to monitor both hierarchical and non-hierarchical plans.

#### 8.2.1. Set-Up

A smart office environment has two floors with identical floor plans, as shown in Figure 11. Each floor is equipped with an IoT coffee machine, which is located at waypoint w4. The robot started on the first floor, at location w2 and was tasked with delivering a cup of coffee to a human. Specifically, the robot’s goal was (and (is-full cup1 coffee) (has-object human1 cup1)). This task involved the following high-level steps: (i) getting a cup from location w3 on floor 1, (ii) asking one of the machines to fill the cup and (iii) delivering the cup to a human, located at w5 on the second floor.

We evaluated our framework on three possible situations: (i) the coffee machines always being available, (ii) the coffee machine on the first floor becoming unavailable and (iii) the coffee machine on the second floor becoming unavailable. The produced plans, for when neither coffee machine malfunctions, are provided in Appendix C. For the last two situations, we disable the coffee machine just before the robot reached it, that is, when the robot was executing the drive-base action that would have resulted in it reaching the coffee machine. This was simulated by sending a message to our cloud back-end, informing it about the coffee machine’s unavailability, that is, we acted as the IoT middleware.

The experiment was ran for our hierarchical approach and for when everything is planned upfront (referred to as “No hierarchy” in the results table). In both cases, our framework informed the robot about the coffee machine malfunctioning and, when necessary, replanning was triggered. Only the highest level of the hierarchy differs from the previous experiments. For this experiment, it contains three composite actions: get_object(?robot ?object), go_fill_object(?robot ?object ?filling), give_object_to_agent(?robot ?object ?agent). These action decompose by re-using the object domain (from Figure 9) and its subsequent layers. For the hierarchical planner, a single remote object represents all IoT devices in the environment, that is, the lift and the two coffee machines.

For planning everything upfront, all primitive actions are contained within a single domain file. As the planner requires the location of all the coffee machines upfront, the single remote object cannot be used. Only the 3 remote devices required for the scenario are included within the problem. As shown in Section 8.1, adding more devices would slow down the planning due to an increase in the number of calls to the capability reasoning module.

#### 8.2.2. Results and Discussion

The results, shown in Table 3, present the total planning time, planning time before the first primitive action was executed and number of executed primitive actions. All times are given in seconds and are the average of 5 runs. The total planning time was longer when a hierarchy was used, than without a hierarchy. This is due to the overhead of starting multiple (i.e., 13) instances of the task planner. Nevertheless, with a hierarchy, the robot started acting sooner because the robot, initially, only decomposed the first branch of its plan (see Appendix C, Listing 12) and the navigation layer only reasoned on the waypoints located on the robot’s initial floor (rather than both floors).

When neither coffee machine broke down, our hierarchical approach produced a plan of worse quality, that is, more primitive actions were executed, than the approach without a hierarchy. This was due to the coffee machine on floor 1 being selected, which produced a suboptimal plan. In a different situation/environment this difference could have been even larger. We see two options for improving this in future work. As the cloud’s Plan Validator knows what the robot’s plan is, the cost of a device executing an action could incorporate knowledge about the current and goal states of the robot. Alternatively, as the robot is informed about the location of both devices, it could create a plan to use each of the devices and select the shortest one. Both these approaches require further investigation.

When the coffee machine on floor 1 became unavailable, with our hierarchy the robot replanned the layer containing the remote device and in total executed 20 primitive actions (see Table 3). Planning without a hierarchy was not affected by coffee machine 1 breaking down as its plan did not contain that device. In contrast, for planning without a hierarchy, when the coffee machine on floor 2 became unavailable, the robot executed 32 actions as it navigated back to floor 1. These results demonstrate that even though planning everything upfront guarantees completeness, in dynamic environments it can be beneficial to deploy a hierarchical approach.

Moreover, if the coffee machine becomes unavailable and the robot has not started to execute the move_to_object(robot1, remote) action (i.e., is still fetching the cup), no replanning is performed by our hierarchical method. In contrast, for planning everything upfront, replanning is still required. Furthermore, if the coffee machine becomes available again, replanning was pointless. This is also applicable to any state change that may occur, for example, the planned cup being used by another agent, a door being closed, the human moving location and so forth.

### 8.3. Exp. 3: Comparison with Planning Everything Upfront

In order to compare the computational times of planning everything upfront and our hierarchical method, we present the planning times for differing environment complexities. By incrementing the number of floors in our scenario, we are able to increase the complexity of the environment at a steady rate, that is, the total number of object rises linearly. These objects include: waypoints, doors and all IoT devices. As an example, a floor is displayed in Figure 11.

#### 8.3.1. Set-Up

For this experiment the robot navigated from its start location (i.e., waypoint1.1_room1.0) to a room on a different floor, which was chosen at random. The plans produced when the randomly selected room is room2.2, for planning everything upfront and for hierarchical planning are shown in Listings 6 and 7, respectively. We selected 3 random different rooms and ran each experiment 5 times; thus, each point on the results graph is the average of 15 runs.

The planning everything upfront approach takes, as input, a single domain file containing the actions required to navigate a multi-floor environment, that is, drive_base, request_lift, request_floor and open_door. The State Estimators populate the problem file with the initial state for the following objects: a lift, the local robot, all remote devices (e.g., IoT lift and IoT doors), doors, floors, rooms and waypoints; and the goal is set to the required room, that is, (exists (?w - waypoint) (and (at-base ?w rob1) (in-room ?w <the required room>))).

For our hierarchical approach, we opted to reuse the multi-floor navigation domain and navigation domain (shown in Figure 9). The goal for the multi-floor navigation layer is set to the required room, that is, (at-location rob1 <the required room>). This layer contains state for rooms, floors, lifts, the local robot and a lift device. As shown in Listing 7, this will plan the use of the navigate_to_location composite action, which uses the navigation domain. The navigation problem file contains the objects (i.e., rooms, doors, the local robot, door devices and waypoints) and state for a single floor. Its goal is set to, for example, (exists (?w - waypoint) (and (at-base ?w rob1) (in-room ?w <the required room>))).


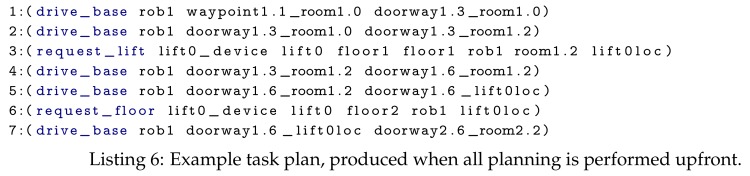



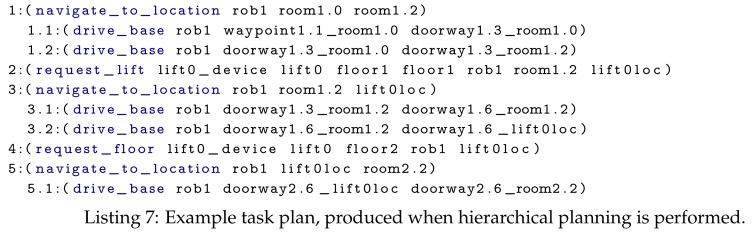


#### 8.3.2. Results and Discussion

**Figure 12:**
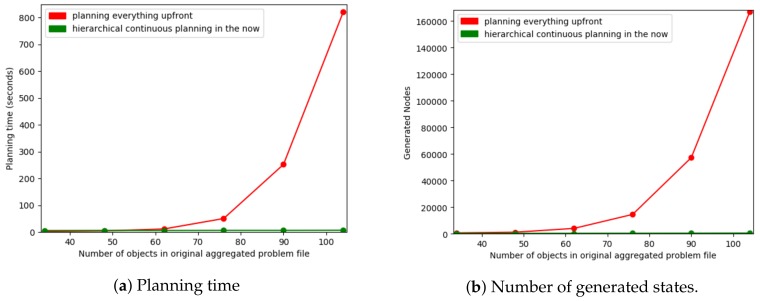
Planning times for planning everything upfront (red line) versus using a hierarchy (green line). For each result three random rooms were selected as goal locations and all experiments were ran 5 times.

As we increase the complexity of the environment there is an exponential increase in the planning time, this is shown in Figure 12a. Figure 12b shows the number of states TFD/M generates as this is deterministic and thus is not hardware dependent and does not vary between runs. For simpler environments, for example, less than 48 objects (split in 3 when our hierarchy is used), planning everything upfront performs slightly better than our hierarchical planner. This is due to the overhead of starting multiple instances of the planner in order to produce the layered plan. As planning everything upfront resulted in a much steeper exponential increase in time, our hierarchical approach performs substantially better in more complex environments. Our hierarchical planner only inserted objects on the robot’s current floor into the problem of the navigation layer, allowing the planner to reason over less unnecessary information (and thus fewer states). This splitting up is also applicable to many other domains, for example, a factory domain, in which the knowledge about the different items being constructed can be split-up or a kitchen environment for which different meals (such as, making coffee or making a cheese sandwich) can be split-up.

### 8.4. Exp. 4: Varying the Number of Levels in the Hierarchy

How the PDDL actions and state knowledge are split-up has a large impact on the planning time. In comparison to planning everything upfront, our approach decreases the planning time for large state spaces. In contrast, for small state spaces our approach increases the planning time. Experiments presented in this section show how much of an impact adding more layers has on the computational cost.

#### 8.4.1. Set-Up

To show results for when deploying a hierarchy produces longer and shorter total planning times we chose two environment complexities: (i) 70 objects split across 4 floors and (ii) 130 objects split across 8 floors. These experiments use the scenario and each of the actions and state knowledge splits described in Section 6, that is, the robot findings out what the child’s request is, then accomplishes that request (which is to switch on a night light). This scenario could also be applied to fulfilling requests in a multi-storey office environment or a smart hotel. Moreover, rather than splitting the knowledge by floor, in a different scenario, we could have split by, for example, the objects required for different tasks. We choose floors for our experiments as it is reasonable that the the total number of object rises linearly when the number of floors is increased and to not overcomplicate this paper with many different hierarchies.

For these experiments, the State Estimators populated the problem file with the initial state for the following objects: a lift, the local robot, all remote devices (i.e., an IoT lift, all IoT doors), doors, floors, rooms and waypoints. Both the robot and child are located on different floors. As before, all experiments were ran 5 times. Each experiment employed a set of domain files and produced the corresponding plan, described in Section 6 (and Appendix B). The “DF1” experiment used one domain file, containing all actions and thus everything was planned upfront (i.e., Figure 6). For “DF2” the two domain files and the executed actions are shown in Figure 7; the domain files and executed actions of “DF3” and “DF4” are shown in Figure 8 and Figure 9, respectively.

#### 8.4.2. Results and Discussion

As shown in Table 4, for 70 objects (4 floors) the more we split-up the actions and state the longer the total planning time. On the other hand, when our fully hierarchical approach (i.e., DF4) was ran, the first primitive action was executed sooner than when everything was planned upfront (i.e., DF1). When there were 130 objects (8 floors) the total planning time was shorter for our fully hierarchical approach. This was especially true for the amount of time spent planning before the first primitive action was executed.

## 9. Conclusions and Future Work

In this paper, we presented our Hierarchical Continual Planning in the Now framework, which aims to improve a robot’s ability to act in dynamic environments with the assistance of IoT devices. IoT state knowledge is monitored by components in a cloud-backend and the robot is only informed about state relevant to its current task plan. During the planning phase, a single remote device object represents all remote (IoT) actuators; therefore, the robot’s plan is less likely to require replanning when a devices becomes unavailable. Further, our experiments showed that when the environment contained 10 IoT devices, the number of calls to the external capability checking module decreased by 92%.

World state further in the planning horizon is harder to predict and more likely to change. Therefore, tasks are split-up into subtasks and the first subtask is planned and executed before the subsequent one. Planning time increases exponentially as the number of objects increases [45,46]; thus, to keep planning times tractable, only state relevant to the robot’s current context is inserted into the planning problems. In our coffee bot scenario, these features enabled the robot to start acting after 3.68 s rather than 11.30 s. Conversely, planning everything upfront guarantees completeness and, when no replanning is required, the plan is likely to be more optimal than when a hierarchy is used. Nevertheless, our framework reduces the need for a robot to frequently replan and when replan is required, only a single branch (i.e., subtask) is replanned.

We see several directions for future work. First, our approach may lead to suboptimal plans. For example, if a robot is tasked with fetching a cup of coffee it would plan three distinct subtasks, that is, 1. collecting a cup, 2. filling the cup using a coffee machine and 3. delivering the coffee. Which cup is more efficient for the robot to collect may depend on the location of the coffee machine but currently this is not taken into consideration while collecting the cup. In the future, we will investigate how information about the subsequent composite actions can be integrated within the composite action currently being executed.

Second, we make a worst-case assumption during planning, that is, that the world will constantly change in heterogeneous ways and these changes will continuously cause the robot’s task plan to become invalid. In the future we will investigate applying the “just in time” concept to our planner, which will minimise the time between executing each branch of a plan. This entails planning a subsequent branch while the current branch is still being executed.

Third, we intend to evaluate running a probabilistic planner (e.g., Reference [48]) to handle uncertainty in the outcome of actions. In particular we think this could be beneficial to our lower-level actions, such as navigation and object manipulation. Combining observations from noisy IoT and on-board sensors could help determine the probability of the environment’s state, for example, the robot’s or object’s position.

Fourth, our architecture could be extended to multi-robot systems requiring conflict resolutions, that is, in which multiple robots require the same resources to complete their plans. One possible solution would be to expand our cloud-based Plan Validator. This component receives a copy of all robots’ plans; therefore, could search for conflicts and inform robots about restrictions they must adhere to. Resolving conflicting states without a centralised planner is likely to result in a suboptimal solution and if the robot cannot adhere to the provided restrictions, its planning will fail. Moreover, a robot could unset the state required by another robot, for example, close a door the another robot has just opened. How such challenges can be overcome by our system requires further investigation. For instance, the Plan Validator could contain a planning component that attempts to merge the robots’ plans, then informs the robots of the modifications made to their plans.

Last, the framework could incorporate the functionality to learn about what actions/tasks have failed and what the state of the environment was at that time. As humans tend to have daily routines, this could include knowledge about what time of day the action was executed. This knowledge can be processed to tune the cost of actions (e.g., increase the cost of actions likely to fail) and to improve the state estimation.

Merging IoT sensors and actuators and robotics is a promising trend in distributed robotics. We hope that our paper may inspire other researchers to further work in this emerging domain.

## Figures and Tables

**Table 1:** Description of the different State Estimators.

State Estimator	Description
object_state	The object_state estimator is configured with a list of objects (given as arguments at initialisation or added later by calling a method) that the planner requires the state of. The objects’ state is read from the robot’s knowledge base and the relevant statements are added to the planner’s current state. A statement is relevant if the predicates and object types, contained within the statement, have been defined in the domain file. When one of its arguments is “request”, then all requests sent (by the cloud) to the robot are inserted into the PDDL problem file. This enables a human to request the robot’s assistance at any point in time.
locations	This Static State Estimator inserts the waypoints, which are within the robot’s knowledge base, into the PDDL problem. Waypoints are written in the format *<ID>_<roomID>* (e.g., doorway1.2_room1.1) and those with matching <ID>s (e.g., locations either side of doorways) are set as being in-line.
robot_pose	Obtains the robot’s position from odometry and localisation. If the position is equivalent to a location that has previously been added to the state, the robot is assigned to the location using the at-base PDDL predicate. If the robot is at an unknown location, a new location is created and inserted into the PDDL problem file. Based on the work of Reference [3].
sensed_obstacles	Adds PDDL statements for any identified obstacles on the robot’s path. Identification can come from running object recognition algorithms on a robot’s RGB camera or directly from the cloud (e.g., closed door). Obstacles that have been acted on and are no longer obstacles, are removed from the problem.
goal_creator	Sets the robot’s goal. This Static State Estimator is used at the highest level of the hierarchy. Further details are specific to the experiments that were ran (see Section 7 and Section 8).

**Table 2:** Comparison of adding all devices to the PDDL problem versus using a single object to represent all remote devices. “Generated States” is the aggregated number of states TFD/M generated during its search for the (sub)plans.

	All Devices Represented					Single Device Represented
**Number of Devices**	**5**	**10**	**15**	**20**	**25**	**Any**
**Calls to capability module**	365	680	1005	1520	1850	56
**Generated States**	749	1035	1346	1913	2241	349

**Table 3:** Comparison of planning times and number of executed primitive actions for the fetching coffee scenario. *t* is the total planning time, *t’* planning time before the first primitive action is executed, *e* is number of executed primitive actions. All times are given in seconds and are the average of 5 runs. “No hierarchy” was not affected by coffee machine 1 breaking and “Hierarchical” was not affected by coffee machine 2 breaking.

	No Device Breaks		Coffee Machine 1 Breaks		Coffee Machine 2 Breaks	
	Hierarchy	No Hierarchy	Hierarchy	No Hierarchy	Hierarchy	No Hierarchy
***t***	11.30	5.27	13.68	-	-	8.21
***t*’**	3.68	5.27	3.59	-	-	5.09
***e***	19	15	20	-	-	32

**Table 4:** The impact increasing the number of layers has on the number of states and the planning time, when there are 70 and 130 objects in the original aggregated PDDL problem file. *t* is the total planning time in seconds, $t^{\prime }$ the planning time before 1st primitive action was executed and *s* is number of generated states.

	70 Objects				130 Objects			
	**DF1**	**DF2**	**DF3**	**DF4**	**DF1**	**DF4**	**DF3**	**DF4**
$t^{\prime }$	10.66	6.05	7.10	6.14	77.07	21.82	15.64	6.52
*s*	2537	1126	1034	347	13,045	5905	4684	413
*t*	10.66	12.46	13.95	14.93	77.07	53.89	47.70	16.33

## Supplementary Materials

The following are available online at https://www.mdpi.com/1424-8220/19/22/4856/s1, Video S1: planning in a smart home—open blinds, Video S2: planning in a smart home—switch on light.

## Abbreviations

The following abbreviations are used in this manuscript:

IoT	Internet-of-Things
IoRT	Internet-of-Robotic-Things
OWL	Web Ontology Language
SRDL	Semantic Robot Description Language
PDDL	Planning Domain Definition Language
FD	Fast Downward
TFD/M	Temporal Fast Downward with Modules
HTN	Hierarchical Task Network
MDP	Markov Decision Process
BDI	Belief-Desire-Intention
HPN	Hierarchical Planning in the Now
DYAMAND	DYnamic, Adaptive MAnagement of Networks and Devices

## Appendix A. Description of Actions

A description of the composite and primitive actions, used for our experiments, are provided in this section.

### Appendix A.1. Composite Action Description

The Composite Action Executors are described in Table A1.

**Table A1:** Description of composite actions.

Composite Action	Description	Example Goal(s) for Lower-Level
discover _request	The robot must move to the human who has a request and ask them what they want. Uses the object domain file to generate a plan.	(not (is-unknown request1))
perform _request	The goal, which is read from the robot’s knowledge base, of its task planner instance will depend on the request. The effect of this action is: (is-completed ?request). In our example scenarios the object domain is used; however, a different domain could be used for differing requests.	Depends on what the request is. e.g., (is-on night_light)
recharge	Robot needs to navigate to the nearest waypoint that is a charging location. Uses the multi-floor navigation domain.	(at-location rob1 waypoint1_room1.1)
move_to _object	Gets the location (i.e., room or waypoint) of the object (e.g., human) from the knowledge base. Creates an abstract plan for the robot to navigate to the correct floor and room using the multi-floor navigation domain.	(at-location rob1 waypoint2_room2.0) or (at-location rob1 room1.1)
guide_to _location	This is similar to move_to_object but an agent (e.g., human) is following the robot to the location. Creates a plan for the robot to navigate to the correct floor and room, using the multi-floor navigation domain.	(at-location rob1 waypoint2_room2.0) or (at-location rob1 room1.1)
navigate _to_location	Its plan will contain actions for the robot to navigate to from a specific waypoint to a location on the same floor as the robot. Uses the navigation domain.	(at-base rob1 doorway1.1_room1.1) or (exists (?w - waypoint) (and (at-base ?w rob1) (in-room ?w room1.1) )

### Appendix A.2. Primitive Action Description

The Primitive Action Executors are described in Table A2.

**Table A2:** Description of primitive actions.

Primitive Action	Description
identify_required _object	This ask is planned when there is an unknown PDDL object (e.g., a request), which the robot requires another agent (i.e., human or remote device) to identify. Its Action Executor looks up what type of object should be identified from the knowledge base and displays the relevant options to the agent.
drive_base	Calls the move_base ROS ActionLib to allow the robot to navigate the environment. The robot can drive between two locations that are in-line or are in the same room. Based on Speck et al.’s work [3].
pick_up_object	Allows the robot to pick up an object. The Action Executor will determine the details of how to pick up the specific object.
hand_over_object	Enables the robot to give an object (e.g., cup) to another agent (e.g., IoT coffee machine or human).
open_blinds open_window	For all remaining actions in this table, the robot used in our experiments is not capable of performing them; therefore, the action is executed by the RemoteActionExecutor. In our smart home blinds and windows are IoT enabled.
fill_object	Fills an object (e.g., cup) with something (e.g., coffee).
switch_object_on	Used to plan the switching on of an object (e.g., a night light).
request_lift	This action can only be executed when the robot is inside the room with the entrance to the lift. The robot used in our experiments is not capable of pressing the lift button.
request_floor	When the robot enters the lift, it request this action is preformed, to get to the correct floor.
switch_on_room _light	This action, and the open_door action, are added to the navigation domain when required, that is, when a room the robot is about to drive into is dark.
open_door	Enables the robot to open (IoT enabled) doors which are blocking its path.

## Appendix B. Example of the Executed Actions at Varying Hierarchical Splits

The plans produced by our framework, when provided with the PDDL files for each of the design steps (described in Section 6), are displayed in this section. To produce these, the goal_creator set the robot’s goal to (is-recharging rob1). When all actions are contained within the a single domain file, a derived rule is defined, which states that if the robot is at a charging location (e.g., waypoint1.1_room1.0) and all its requests are completed, then (is-recharging rob1) is true. When a hierarchy is used, a condition of the recharge action is set to (not (has-incomplete-requests)); has-incomplete-requests is a derived rule that checks all requests are completed. We chose to do this so that when a new request is sent to the robot, if it is recharging, the recharge action is cancelled. This causes a replanning, which results the robot creating a plan to accomplish the new request and return to a charging location. As mentioned in Table 1, the object_state creator inserts requests into the PDDL problem file.

### Appendix B.1. No Splitting

When all action are included in a single domain file (see Figure 6) the plan in Listing 8 is produced. A precondition of the switch_object_on is that the request has been identified as the correct type, that is, (is-switch-on-request ?request); and one of its effects is set to (is-completed ?request).

### Appendix B.2. Splitting up Key Concepts

As show in Figure 7, the main tasks the robot can perform can be abstracted, enabling the plans of Listing 9 to be produced. When the discover_request action is executed, its Composite Action Executor will use the “primitive domain” to decompose this action into primitive actions. After discover_request has been executed, perform_request, then recharge, are executed by also creating plans containing primitive actions. This split result in the robot being less likely to have to replan, and enables the creation of a (high-level) human understandable plan.

### Appendix B.3. Separate Repeated Blocks

In many domains, the robot will be required to navigate the environment. Therefore, as shown in Figure 8, navigation-based actions can be split into a separate domain file. The example hierarchical plan is shown in Listing 10.

### Appendix B.4. Reducing Unused Knowledge

Rather that reasoning about the whole environment in detail, this information can be split-up. As shown in Figure 9, the “multi-floor navigation domain” enables the robot to plan the high-level steps of its navigation (i.e., request a lift to get to the correct floor and navigate to/from the lift from either its initial location or its desired location), and the “navigation domain” performs the detailed steps required to navigate a single floor. An example hierarchical plan is shown in Listing 11.

## Appendix C. Plans Produced during the Simulated Coffee Fetching Experiment (Exp. 2)

In the experiments described in Section 8.2, a robot is tasked with fetching a coffee for a human. The plans produced when neither coffee machine malfunctions are presented in this appendix. The actions planned when the first primitive action was executed and all executed actions, when our hierarchy was used, are provided in Listings 12 and 13, respectively. When everything is planned upfront the plan of Listing 14 is produced.
